# A Manual of Surgical Treatment

**Published:** 1899-08

**Authors:** 


### Book Reviews.

A MANUAL OF SURGICAL TREATMENT. By W. WATSON CHEYNE, M. B., F. R C. S., F. R. S., Professor of Surgery in King's College, London; Surgeon to King's College Hospital, etc.; and F. F. BURGHARD, M. D. and M. S. (Lond.), F. R. C. S.. Teacher of Practical Surgery in King's College, London; Surgeon to King's College Hospital, etc. In six imperial octavo volumes, with illustrations. Vol. I, 285 pages, with 66 illustrations. Philadelphia and New York: Lea Brothers and Co. 1899.

The authors of this work have done just what the medical profession has been waiting for these many days. They have written a manual of surgical treatment, dealing chiefly with conditions, not theories, and giving information of practical value, not contenting themselves with telling what might be, but pointing out clearly and distinctly what is, and then describing what is best to do and when and how and why to do it.

A most noticeable feature of the first volume just issued is the absence of pathology. It would be unwise for a modern practitioner to feel satisfied with this work alone. It is a complete working volume, certainly, but a side knowledge of surgical pathology and surgical conditions which are abridged or passed over in this work is absolutely indispensable. Thus a complete, modern and safe work on general surgery belongs by the side of this one. Such a volume as Park's, for instance, in connection with Cheyne and Burghard would be all that any intelligent man would absolutely need in the way of surgical works. Park is needed for its inestimable value in tumors and intestinal surgery, and above all for the very excellence of its surgical pathology; Cheyne and Burghard for its confidential way of telling of surgical conditions and their treatment.

In their introduction the authors say: "The great essential is full and detailed information as to the best methods of treatment. We have ourselves frequently experienced the want of detail, especially as regards the further treatment of our cases, and have had to learn the best methods of procedure from experience. It is this want that the present work is intended to supply. . . . We have tried to put ourselves in the place of those who have to treat a given case for the first time, and we have endeavored to supply them with details as to its treatment from the commencement to the termination of the illness. We have assumed that the writer is familiar with the diagnosis of the disease, and we only refer to the pathology and symptoms in so far as it is necessary to render intelligible the principles on which the treatment is based and the various stages of the disease to which each particular method is applicable."

That, in brief, is the prevailing idea. It is really a post-graduate book. No frills, no garnishing, no side issues. Nothing at all but meatiness. Just plain talk, in which every sentence counts. The authors write like men who have themselves felt the need of just such a book as they have given the profession—a working book, and to the end that it may be easily handled the publishers have put it into six volumes. Thus each volume is small; it is compact, it is fruity and it is handy. The day of lumbering, dead-looking and stalky medical works has passed. Hard as it is to find time to keep abreast of progress in these rush-days, it is still harder to pore over an immense thick book, which possibly is a nineteenth century fifth edition of an ante-bellum days first edition, republished because its author has been forced by progress of events to dress up the old introductions, add a little here and there on new subjects—sort of polish up the front door knob as it were—and because the publishers had the plates handy. The doctor of today wants a book that he can slip into his pocket if need be and read at odd times; he wants a book that he can catch up between times, drop into an easy chair, lean back and read and be comfortable. That's just the kind of a work Lea Brothers are putting out for Cheyne and Burghard. Aside from the rare value as a work on surgery it is a cosy sort of a book in itself.

In the first volume the opening chapters are devoted to inflammation, suppuration, and gangrene and ulceration. Under a general heading of wounds comes the management of operation, dangers of operation, after-treatment and mode of healing. The various varieties of wounds are taken up and handled by the method and in the manner indicated in the extract from the introduction.

Syphilis, chancroid, tuberculosis and tumors comprise the closing chapters. One of the most valuable chapters in the work, and one of immense importance is that on anesthesia and the giving of anesthetics by Dr. Fred J. Silk. This is really the most practical and understandable chapter on anesthesia which has been published in a long time, for Dr. Silk goes into detail and gives reasons for what he does, taking up each anesthetic—general and local, and advising as to use, in different conditions. There are no pretty pictures in this book. What illustrations there are tell some thing.

The volumes to follow will cover the subjects in this order: Part II., The treatment of the surgical affections of the tissues, including the skin and subcutaneous tissue, the fasciæ, bursæ, muscles, tendons and tendon-sheaths, nerves, arteries and veins; deformities. (In preparation.)

Part III., The treatment of the surgical affections of bones and joints, including fractures and dislocations, excisions and amputations. (In preparation.)

Part IV., The treatment of surgical affections of the head, face and neck; throat, nose and ear. (In preparation.)

Part V., The treatment of the surgical affections of the thorax, breast, spine, and generative organs. (In preparation.)

Part VI., The treatment of the surgical affections of the alimentary and urinary systems. (In preparation.) N. W. W.

